# Global hinge sites of proteins as target sites for drug binding

**DOI:** 10.1073/pnas.2414333121

**Published:** 2024-11-25

**Authors:** Haotian Zhang, Mert Gur, Ivet Bahar

**Affiliations:** ^a^Department of Computational and Systems Biology, School of Medicine, University of Pittsburgh, Pittsburgh, PA 15261; ^b^Laufer Center for Physical and Quantitative Biology and Department of Biochemistry and Cell Biology, School of Medicine, Stony Brook University, New York, NY 11794

**Keywords:** Gaussian Network Model, protein dynamics, drug binding sites, ensemble analysis, collective motions

## Abstract

Hinge sites of proteins play a key role in mediating conformational mechanics. Among them, those involved in the most collective modes of motion, also called global hinges, are of particular interest, as they support cooperative rearrangements that are often functional. Yet, the utility of targeting global hinges for modulating function remains to be established. We present here a systematic study of a series of proteins resolved in drug-bound forms to examine the probabilistic occurrence of spatial overlaps between hinge sites and drug-binding pockets. Our analysis reveals a high propensity of drug binding to hinge sites compared to random. Notably, one-third of currently approved drugs are colocalized with hinge sites. These mechanosensitive sites are predictable by simple models such as the Gaussian Network Model. Their targeting thus emerges as a viable strategy for developing a new class of drugs that would exploit and modulate the target proteins’ intrinsic dynamics, and potentially alleviate drug-resistance when used in combination with orthosteric or allosteric drugs.

## Significance

The identification of target sites on proteins, not only the target proteins themselves, is crucial for designing effective modulators of function. The most widely used target sites are the chemically active sites, also known as orthosteric sites. More recently, targeting of allosteric sites emerged as a useful strategy. In the present study, we demonstrate by a systematic study of a large ensemble of drug-bound proteins that a third group of target sites, hinge sites coordinating the global motions of the protein, can serve as alternative target sites for binding drugs. The combined use of drugs targeting such mechanosensitive sites with orthosteric, or allosteric drugs may open alternative avenues for polypharmacological interventions.

Identification of druggable sites is essential to the success of drug discovery. Binding of small molecules to these sites should ensure not only sufficiently high affinity but also effective interference with the target protein function. Orthosteric sites usually meet this requirement, but studies in the last two decades also pointed to the utility of targeting allosteric sites (1–6). Allosteric sites present the advantage of selectively interfering with a specific protein–protein interaction and associated cellular pathway, rather than completely obliterating the protein function. Allo-targeting may be important for selectivity, as subtypes within a protein family often share the same orthosteric site features, whereas allosteric sites are subtype-specific. Polypharmacological strategies that simultaneously target the orthosteric and allosteric sites emerged, toward circumventing drug resistance, exemplified by the allosteric inhibition of Abl kinases (7–9).

In search of potent sites that bind modulators of function, numerous computational methods have been developed. These range from simple grid- and geometry-based cavity identification (e.g., FPocket) (10) and pocket comparison (11) to advanced strategies that consider the protein conformational fluctuations (12, 13) to detect hot spots or cryptic sites (14) or to machine learning algorithms (e.g., Deepsite (15) and P2Rank (16)). Significant efforts are deployed for identifying high-affinity sites and developing scoring functions that discriminate such sites (17, 18). However, high binding affinity does not necessarily mean a high impact on function. In fact, among the multitude of druggable sites identified on a given target protein, it is essential to distinguish those that play a role in defining the functional dynamics of the proteins, not only serving as high-affinity sites. This led to the use of additional filters for extracting “essential” sites from among druggable sites (19–21). Essential sites comprise three groups: (1) chemically active (e.g., catalytic), (2) allosteric, and (3) mechanically sensitive (e.g., hinge-bending) sites that support the cooperative movements of the protein. The 1st group has been the natural target of most approved drugs; the 2nd has garnered attention mostly in the last decade. The 3rd, on the other hand, has not been explicitly exploited, even though numerous studies show the functional significance of hinge motions (22–26).

In many instances, the chemically active sites colocalize with the hinge site enabling a mechanochemical action (27): Nonnucleoside reverse transcriptase (RT) inhibitors (NNRTIs) bind the interface between the fingers (and thumb) and palm subdomains of the DNA polymerase domain, thus interfering with the hinge flexibility that would otherwise enable the RT to “hold” the RNA to be reverse transcribed (28–30). Ruxolitinib, a selective inhibitor, distinctly binds to the ATP-binding cavity of c-Src, with its pyrrolopyrimidine rings strategically aligned toward the hinge region (31). Similarly, the ATP binding site of p38α, a hotspot for drug binding, is coupled to a glycine-rich hinge (32); or Gefitinib, a 4-anilinoquinazoline inhibitor, which targets the ATP-binding pocket of EGFR, forms an exclusive hydrogen bond at the hinge region (33). Notably, ATP competitive inhibitors of kinases generally form hydrogen bond interactions with a central hinge site, as recently reviewed (34), and noted in a systematic study of 2,915 ligand-bound kinase complexes (35). More recently, ACE2 catalytic cleft has been shown to undergo hinge-like movements coupled to the interface that binds to the SARS-CoV-2 spike RBD, suggesting that RBD binding might function as a regulator of ACE2 enzymatic activity (26). These observations suggest that targeting hinge sites could be a viable approach for modulating protein function. However, the general utility of this strategy is yet to be confirmed.

Toward this goal, we explored in a systematic (quantitative) manner the occurrence of overlaps between drug-binding sites and global hinge sites. We considered a dataset of 20 widely studied target protein families and their structural homologs, comprising 7,754 proteins that have been structurally resolved in drug-bound forms. We found that a striking 32.53 ± 10.39% of drug-binding sites colocalize with hinge regions. Further assessment of the statistical significance of this spatial overlap demonstrated hinges are enriched by a factor of 4.13 within drug-binding sites, compared to other sites. These results suggest that the mechanical role of hinge sites may be exploited for designing new drugs.

## Results

### Dataset.

The dataset of protein families considered in the present study is presented in Table 1. The set comprises 7,754 proteins belonging to 20 families. To select these families, we considered the most frequently targeted families of proteins, as reported in a comprehensive mapping (36) of molecular drug targets. Among them, we considered the top-ranking 12, targeted by more than 2/3 of compounds listed in ChEMBL, mainly the GPCR, nuclear receptor, ion channel, reductase, transporter, kinase, lyase, transferase, phosphodiesterase, protease, hydrolase, and cytochrome P450 families. These “major” families included diverse groups/subfamilies/families of proteins with different folds, oligomerization states, and sizes. We selected one to four representative groups (hereafter referred to as families) from each of the 12 major families *SI Appendix*, *Supporting Methods*, which led to 20 families characterized by distinctive UNIPROT groups or functional classes, as listed in Table 1. The protein sizes vary as 159 ≤ *N* ≤ 1,138, where *N* is the number of residues. For each family, a Protein Data Bank (PDB) (37) structure is adopted as reference for sequence and structure alignments and visualization. The number of family members, *m*, varies as 10 ≤ *m* ≤ 1,645 with the smallest and largest memberships corresponding to penicillin acylase (PA) and carbonic anhydrase (CA). The average RMSD of family members from the reference and their SD varies from 0.60 Å ± 0.28 (HIV-1 protease) to 9.78 Å ± 7.92 (sodium channel Nav1.5 a subunit). More information on the dataset selection and properties can be found in *SI Appendix,* Tables S1 and S2 and Fig. S1 *A* and *B* show the distributions of protein sizes (*N*) and family memberships (*m*).

**Table 1.** Dataset of protein families and their characteristics (^*^)


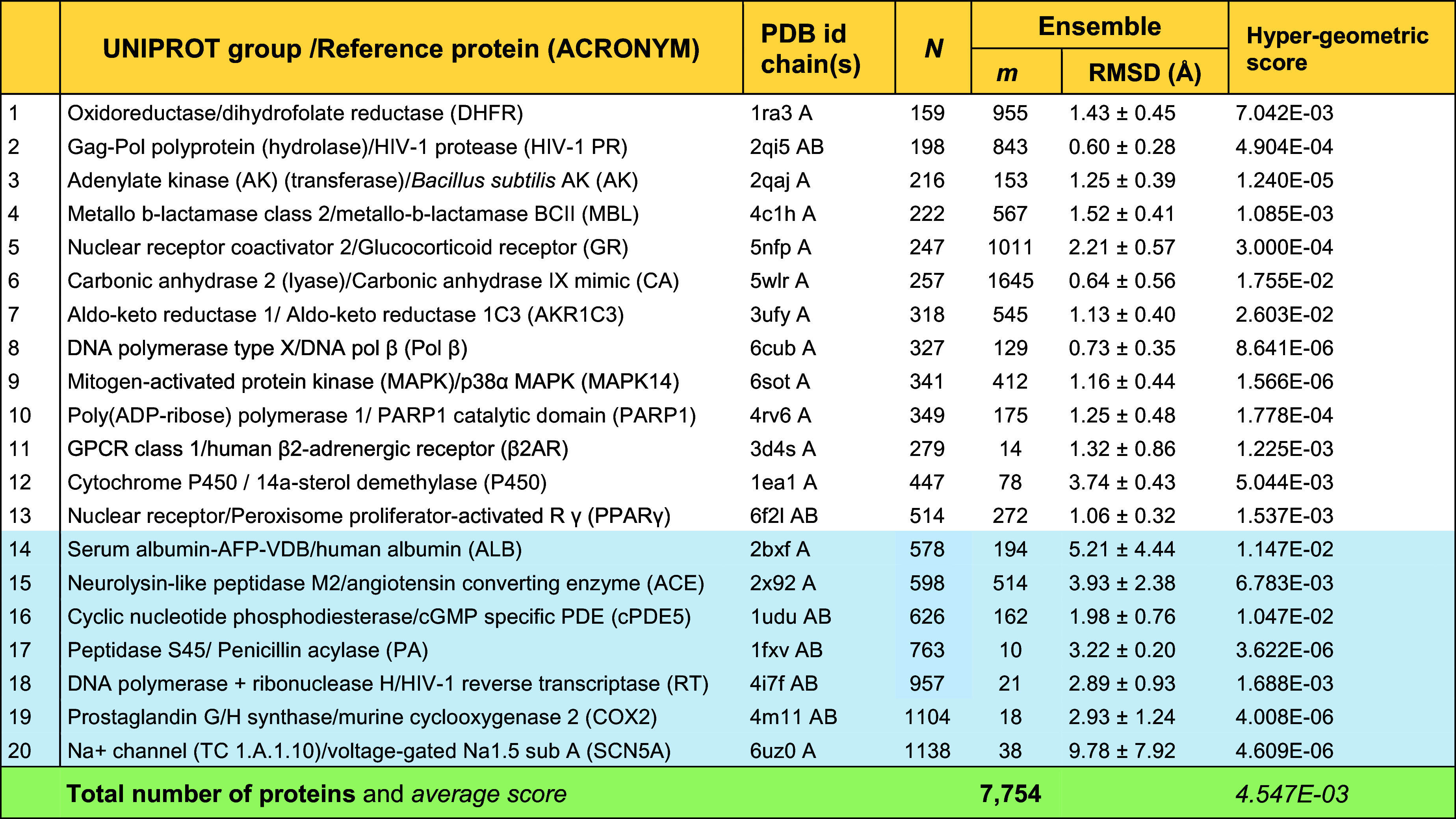

(*) Proteins are ordered in ascending size; N = number of residues; A, B: chain identifiers in the PDB. The RMSD measures the structural similarity with respect to the reference structure. The hypergeometric score provides a metric for the random probability of occurrence of the overlaps observed between hinge and drug-binding residues.

### Approach.

**Fig. 1.**
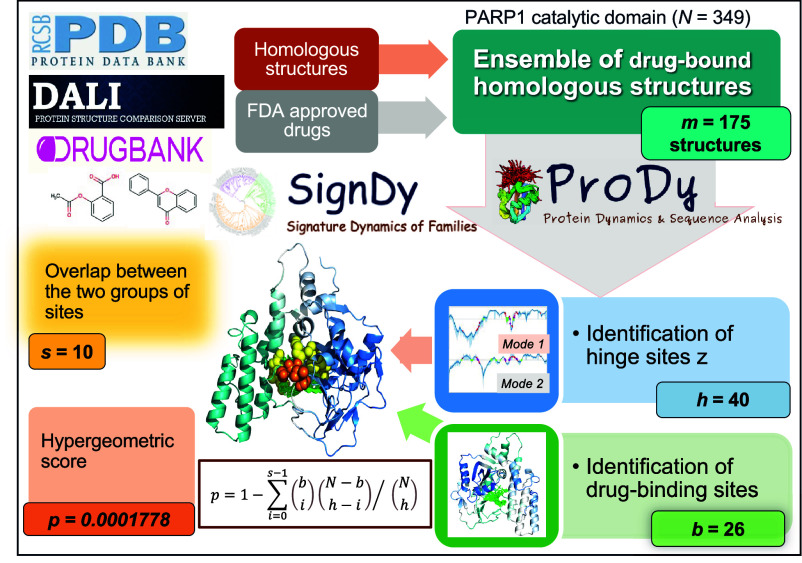
Schematic description of the methodology for evaluating the overlap between global hinge sites and drug-binding sites. Data for PARP1 catalytic domain are shown for illustration. Gaussian Network Model (GNM) analysis yields *h* hinges in modes 1 and 2, *s* of which overlap with the *b* drug-binding residues. The ribbon diagram shows in space-filling the hinge residues in mode 1 (*orange*) and 2 (*yellow*) along with drug-binding sites (in *green;* Fig. 2).

The approach is schematically depicted in Fig. 1. Using the reference structure, an ensemble of *m* drug-bound structural homologs are extracted from the Dali server (38) to identify two subsets of residues: i) those participating in hinge sites that control the signature dynamics of the family and ii) those coordinating the drugs resolved in those structures. Rather than considering individual structures, we adopted the *SignDy* (39) module of *ProDy* (40), to obtain robust information on shared hinge sites and consolidated data on drug-binding sites. *SI Appendix*, Fig. S1*C* displays the number of binding sites, *b*, as a function of *N*. On average, 9.65% of residues participate in binding sites considering the data for all members (*SI Appendix*, Fig. S1*D*). We verified from DrugBank (41) and the PDB biological assembly database (37) that the small molecules included in the analysis were approved drugs and that the structures represented the biologically relevant assemblies/states.

Hinge sites were determined based on the global modes of motion predicted by the GNM for all members of the ensemble (42). Up to three slowest modes, 1 ≤ *k* ≤ 3, were included to ensure a cumulative variance s of ≥ 1/3(*SI Appendix*, *Supporting Methods**)*. Hinge residues in mode *k* were deduced from the *k*th mode profile (normalized displacement of residues along the *k*th mode axis) as those lying at the crossover between negative and positive motions. *SI Appendix*, Fig. S1*E* shows the average number of hinge residues in mode 1-3 for all protein families and *SI Appendix*, Fig. S1*F* their increase with *N*.

For each ensemble, we evaluated the overlap, *s = b∩h*, between the *b* drug-binding residues and *h* global hinge residues (*SI Appendix,* Table S2). Of interest was to find the probability that such an overlap occurs by chance (randomly) for the examined protein, or how different from random was the probability of observing at least *s* of the *h* hinge residues among the drug-coordinating *b* residues. This probability is given by the hypergeometric score (43)

[1] p(s,N,h,b)=1-∑i=0s-1biN-bh-i/Nh,

where Nh denotes the combination of *N* residues taken *h* at a time. The reciprocal 1/*P* provides a measure of enhancement compared to random, and a hypergeometric score of *P* ≤ 0.05 is usually considered significant. Our analysis yielded an average of < *P* > = 4.547 × 10^−3^ with *P* values varying from 1.566 × 10^−6^ (MAPK) to 2.603 × 10^−2^ (AKR1C3) (Table 1), in support of the significance of the overlap between hinges and drug-binding sites, as elaborated below. Details on method and parameters are in *Methods* and *SI Appendix*, *Supporting Methods*. Below we present the results for a few cases, before proceeding to the generalization of observations.

### PARP1.

**Fig. 2.**
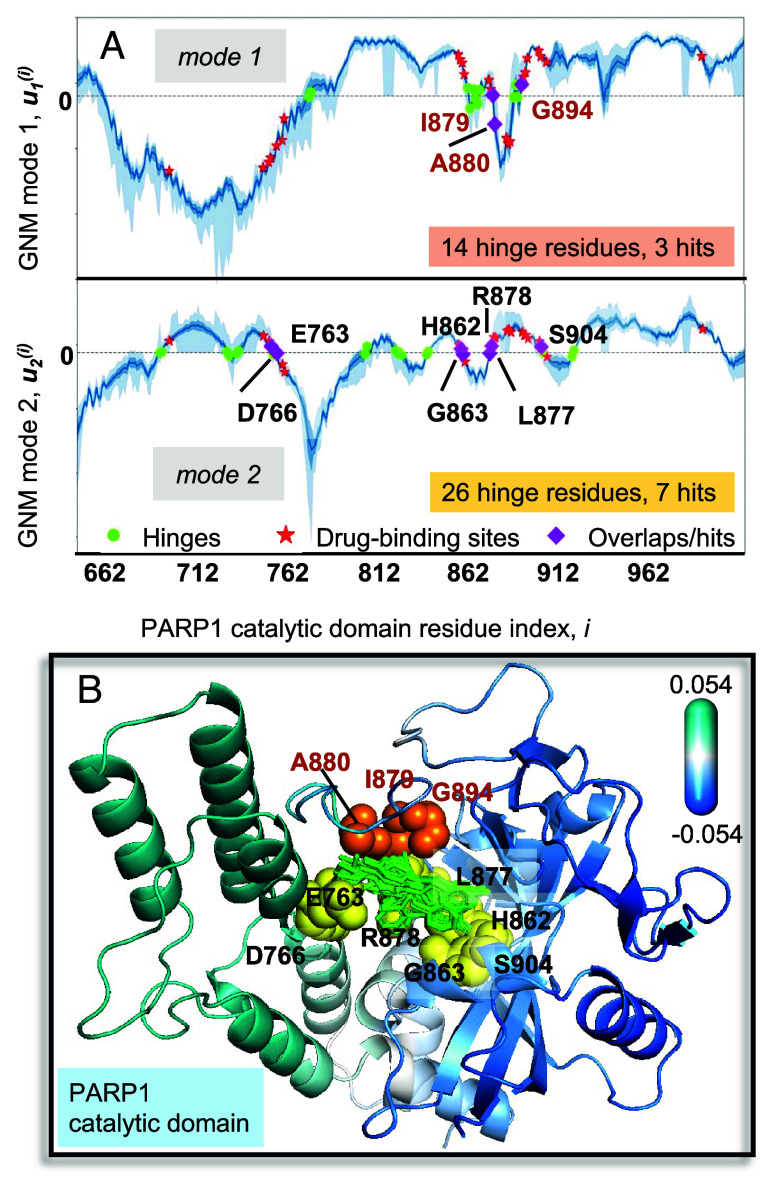
Results for PARP1 catalytic domain ensemble (*A*). Normalized distribution of residue displacements along mode 1 (*Top*) and 2 (*Bottom*) predicted by *SignDy*. The *solid curve* represents the average mode profile, and the *light blue shade* displays the SD based on 175 homologs. Hinge sites (*green dots*), drug-binding sites (*red stars*), and their overlaps (*magenta diamonds, labeled*) are indicated. (*B*) Hinge residues from mode 1 (*orange spheres*), and 2 (*yellow spheres*) line the drug-binding pocket. Drugs from multiple structures are overlaid in *green sticks*. The diagram is color-coded by the motions along mode 1, *blue* and *cyan* referring to opposite direction motions (see *Upper Right scale*).

(Figs. 1 and 2) is a member of the PARP family of enzymes involved in DNA damage repair. Polymerization of Poly-ADP-ribose (PAR) catalyzed by PARP, is essential to signaling the initiation of repair. As such, PARP1 activity has been shown to correlate with longevity. Conversely, PARP1 inhibition is a therapeutic approach against cancer. The results in Figs. 1 and 2 were generated for 175 structures homologous to PARP1 catalytic domain, using as reference the structure resolved in the presence of rucaparib (44). These structures revealed 26 residues that consistently coordinated the drugs: Y710, Q759, A762, E763, D766, L769, E770, W861-S864, L877-A880, V886-G888, G894-F897, K903, S904, Y907, and E988. Among them, I879, A880, and G894 were predicted by the GNM to act as hinges in the first (global) mode of motion intrinsically accessible to the domain (Fig. 2*A*), and E763, D766, H862-G863, L877-R878, and S904 as hinge in mode 2. These two modes were sufficient to satisfy the criterion σ ≥ 1/3 and led to *P* = 1.78 × 10^−4^, with *h* = 40 and *s* = 10. The adoption of three modes yields an even lower *P* value (of 1.350 × 10^−5^) as mode 3 hinges capture five more drug-binding sites. Fig. 2*B* shows the hinge residues at the drug-binding site.

### Angiotensin-Converting Enzyme (ACE).

**Fig. 3.**
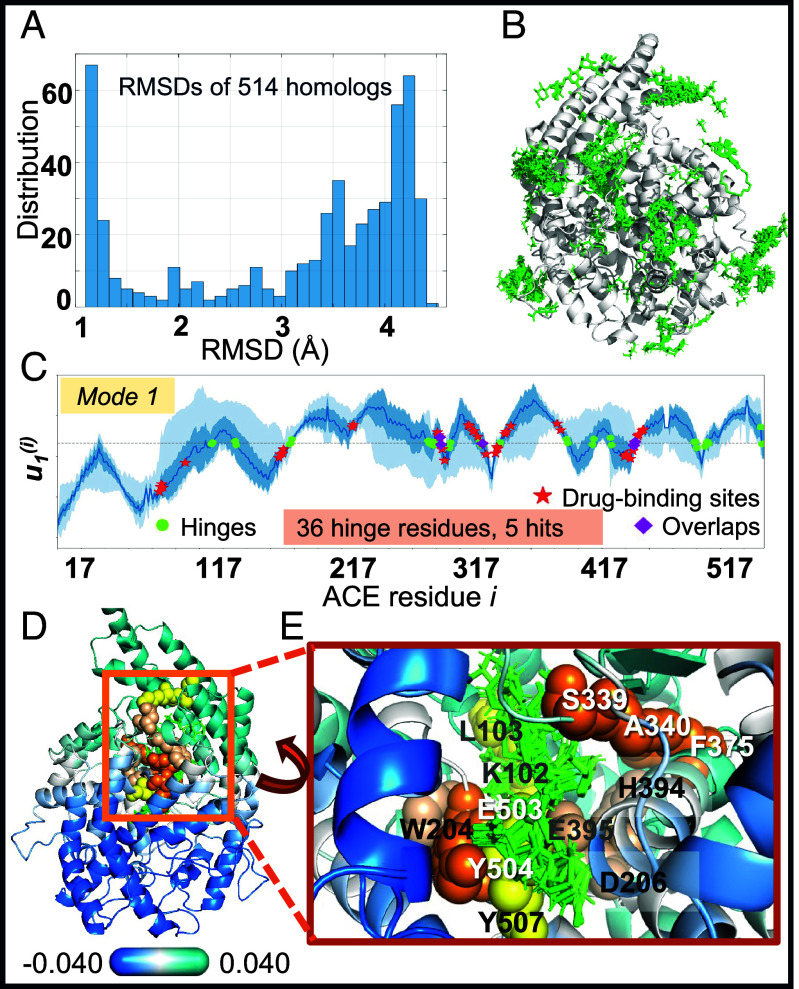
Results for Angiotensin-converting enzyme ACE and its structural homologs. (*A*) RMSD distribution of 514 structural homologs with respect to the reference structure (PDB: 2X92, chain A). (*B*) Positions of small molecules bound to these structures, superposed on the reference structure (*C*) Mode 1 evaluated using *SignDy*. The average curve is in *dark blue*, and the results within one and two SD from the average are indicated by the *dark* and *light blue* shades. (*D* and *E*) Location of hinge residues, and close-up view of the coordination of drugs (superposed from multiple homologs) by these global hinge residues, shown in *orange* (mode 1), *yellow* (mode 2), and *wheat* (mode 3) spheres.

ACE is a hydrolase of *N* = 598 residues acting in the blood pressure-regulating renin-angiotensin system and is a major drug target against hypertension and heart failure. It is composed of two homologous domains, each with an active site, linked by a hydrophobic segment and a glycosylated hinge region. The corresponding ensemble of homologs has 514 drug-bound PDB structures belonging to the neurolysin-like peptidase M2 family, with an average RMSD of 3.93 ± 2.38 Å from the reference structure, the *Drosophila Melanogaster* ACE complexed with ramiprilat (45) (Fig. 3*A*). A diversity of small molecules is found in the ensemble (*green sticks* in Fig. 3*B*). After sorting the drugs among them, *b* = 43 drug-binding residues were identified. The signature dynamics of this family yielded *h* = 80 residues participating in hinge sites in mode 1-3. Fig. 3*C* shows the mode 1 profile which contains 34 hinge residues (*green dots*). Of these, *s* = 12 overlap with drug-binding residues, leading to *P* = 6.78 × 10^−3^. Fig. 3 *D* and *E* display the location of the drugs (from multiple structures) and their coordination by hinge residues.

### Dimeric Targets.

**Fig. 4.**
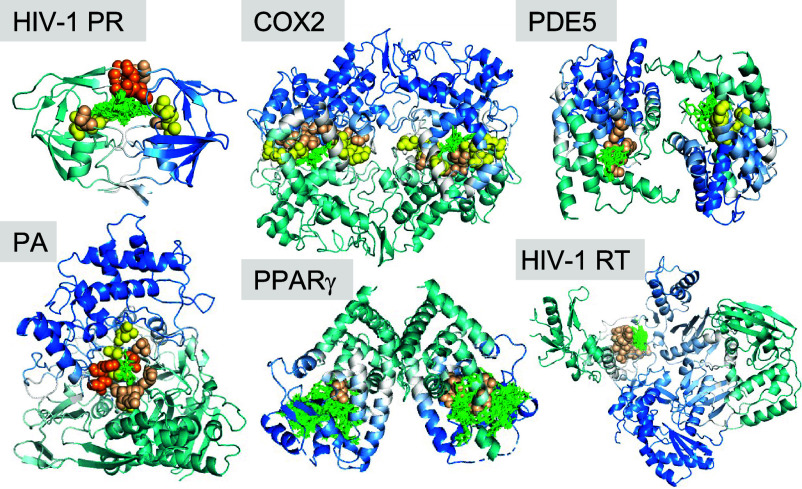
Results for dimeric targets. Dimers may bind drugs at the interface of the two monomers (as in PR and PA) or at active sites within monomers (as in COX2, PDE5, PPARg, and HIV-1 RT). Mode 1 hinge sites, usually located at the interface, are not involved in binding the drugs located within the monomers. Instead, hinges from mode 2 (*yellow*) or 3 (*wheat*) to colocalize with the drugs.

Our dataset had six dimeric proteins (Fig. 4): cyclooxygenase 2 (COX2), cGMP-specific phosphodiesterase (cPDE5), peroxisome proliferator-activator g (PPARg), HIV-1 protease (PR), HIV-1 RT, and PA. Among them, RT and PA are heterodimers; others are homodimers.

Fig. 4 shows that, except for PR, mode 1 hinges (*orange spheres*) do not coordinate the drugs in the homodimers. This is because monomers of homodimers usually undergo symmetric rigid-body movements with respect to each other in mode 1, which allow for their opening/closing or coupled rotations. Therefore, the mode 1 hinge site, at the dimer interface, cannot colocalize with the drug-binding site if the drugs target active sites harbored within each monomer (as is the case for COX2, cPDE5, and PPARg). Hinges mediating mode 2 (*yellow spheres*) and 3 (*wheat spheres*), on the other hand, colocalize with drug-binding sites.

**Fig. 5.**
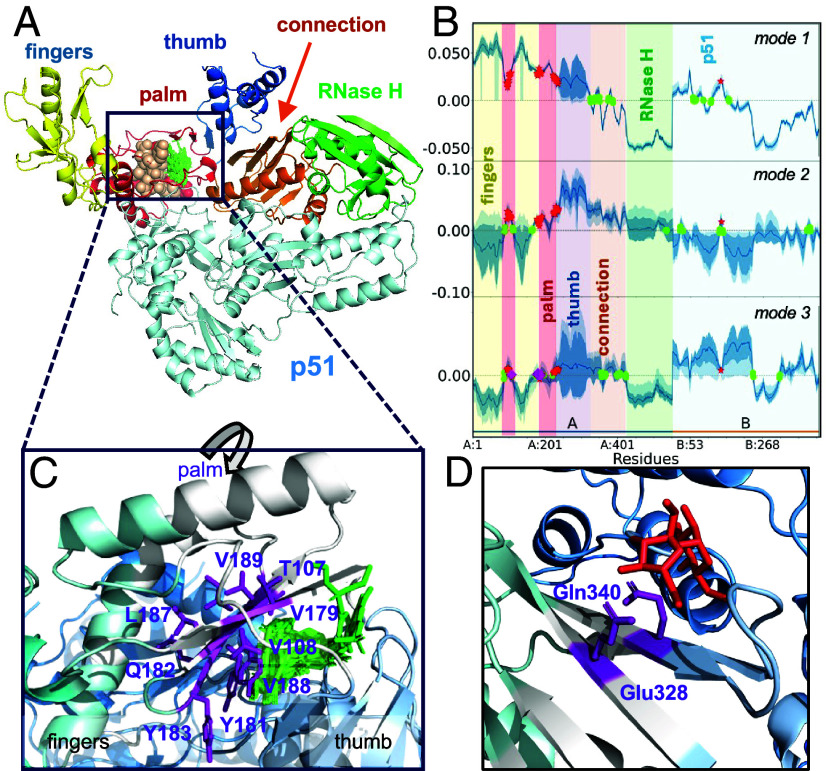
Results for RT ensemble. Panel (*A*) displays the different subdomains of RT p66 subunit in different colors, and the p51 subunit in *light blue*. FDA-approved drugs (*green sticks*) bound to the palm subdomain are coordinated by mode 3 hinge residues (*wheat spheres*). (*B*) RT first 3 mode shapes. The palm is highly constrained in all modes (near *y* = 0 line), as a hinge region. (*C*) Closeup (and rotated) view of interactions at the drug-binding site. Hinges are shown in *magenta*. (*D*) thumb–connection interface also binds inhibitors (*red sticks*). Two hinge residues (*magenta sticks*), identified in mode 1, interact with the inhibitors.

As to the heterodimers, PA binds drugs at the interface between its two subunits, which overlaps with hinge residues from mode 1. In contrast, RT inhibitors (FDA-approved NNRTIs) bind to one of the two RT subunits, mainly the palm subdomain of p66 subunit polymerase domain (Fig. 5*A*). As such, they interfere with the intrasubunit flexing of the fingers and thumb subdomains with respect to the palm. This movement is mediated by mode 3, hence the colocalization of mode 3 hinges (*wheat*) with the drugs at the palm (near V179-E192; Fig. 5*C*). Fig. 5*B* shows the shape of the mode 1-3. The shades separate the p66 polymerase subdomains (palm, fingers, thumb, and connection; see the *vertical labels*) and RNase domain. Other binding sites for inhibitors of RNAse activity have been observed more recently, but not included here as we focused on Food and Drug Administration (FDA)-approved drugs. In particular, the connection subdomain (residues G333-A376 and 388-A400) interfacing with the RNase H domain has been noted to exhibit mutations (46) that confer drug resistance (47). This region is also observed in our analysis to harbor hinge sites (*green dots* in modes 1 and 3; Fig. 5*B*). Furthermore, two residues, E328 and Q340, acting as hinges in mode 1, closely neighbor the inhibitors resolved (48) at thumb–connection interface (Fig. 5*D*).

### A Few Large Targets Are Distinguished by Remarkably High Overlaps.

The *SI Appendix*, Fig. S2 displays the results for 11 additional cases, all monomeric. For relatively larger proteins (starting from serum albumin, ALB, of *N* = 578 residues), highlighted in *blue* in Table 1, we selected more diverse ensembles of structural homologs by relaxing the RMSD threshold (*SI Appendix*, *Supporting Methods*), to have a more complete representation of the conformational space. This resulted in up to 9.78Å average RMSD (for Nav1.5 a-subunit, SCN5A). Notably, SCN5A (of *N* = 1,138 residues) was distinguished by a remarkable overlap between its drug-binding and hinge sites, with a hypergeometric score of *P* = 4.609 × 10^−6^, while its ensemble size was small (*m* = 38). Likewise, COX2 dimer (the 2nd largest protein in our set, with *N* = 1,104) exhibited a highly significant overlap (*P* = 4.008 × 10^−6^) with *m* = 18; and PA (the 4th largest; *N* = 763, *m* = 10) also yielded a *P*-value of the order of 10^−6^. While these large proteins were distinguished by their low *P*-values, the examination of the entire set showed that the correlations of *N* and *m* with *P* values were relatively weak (*SI Appendix*, Fig. S3).

### Overall Results Overlap between Hinge, Drug-Binding, and Functional Sites.

The *P* values for all 20 drug–target families are listed in the last column of Table 1. Other quantitative data (*h, b, s* for all families) are presented in *SI Appendix,* Table S2. The list of hinge residues that overlap with drug-binding sites, also called “hits” can be found in *SI Appendix,* Table S3, broken down by the associated modes (GNM modes 1, 2, and 3). Residue numbers correspond to the reference PDB structure (*column 3* of Table 1). For comparative purposes, we list in *SI Appendix,* Table S3 the functional (e.g., catalytic, ATP-binding, substrate-binding, posttranslational modification) site residues (*column 5*) for each family. The residues in boldface (in *columns 2-5*) are functional residues that are also identified as hinges and drug-binding sites. Notably, the active sites also contain a few other global hinges (written *in blue*). The last column lists the total number of overlaps between the computationally identified hinge residues and the experimentally reported drug-binding and/or functional residues.

## Discussion and Conclusion

### Many Drug-Binding and/or Functional Sites Colocalize With Global Hinges.

The current study presents robust results on the identification of hinge residues and their comparison with drug-binding sites for an ensemble of 7,754 proteins belonging to 20 drug target families. The study demonstrates that global hinge sites often coincide with drug-binding sites (Table 1, *SI Appendix*, Tables S2 and S3) and functional sites (*SI Appendix*, Table S3). The observed spatial/structural overlap between hinge sites and drug-binding sites is significantly higher than expected from random distribution as indicated by the average hypergeometric score < *P* > = 4.547 × 10^−3^. Our analysis further shows that some hinge residues that do not overlap with drug-binding sites coincide with functional sites (noted in *blue* in *SI Appendix*, Table S3), further underscoring the significance of global hinges.

**Fig. 6.**
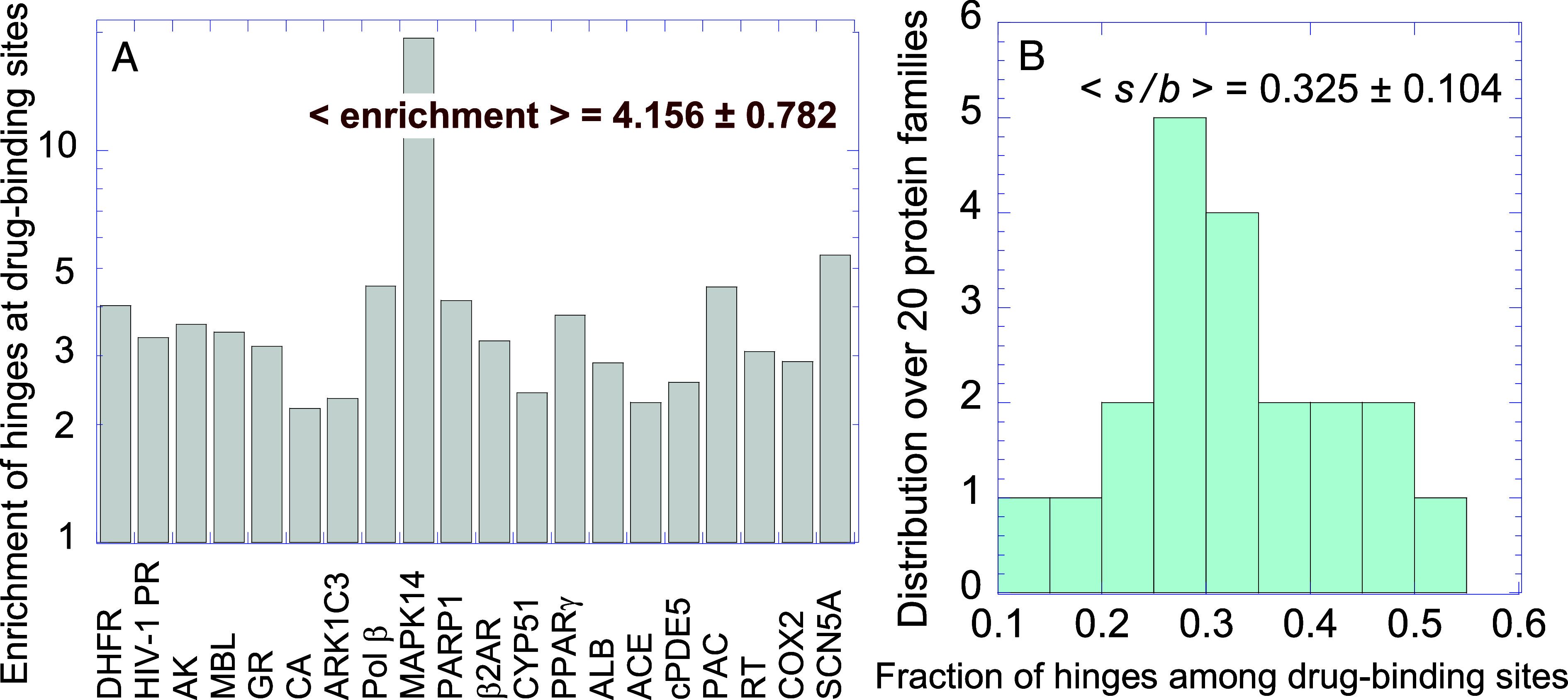
Statistical analysis shows the high propensity of hinges among drug-binding sites. (*A*) Enrichment of hinge residues at drug-binding sites, compared to other regions. (*B*) Histogram of the fraction of hinge residues at drug-binding sites.

As another metric of the degree of overlap between drug-binding sites and hinge sites, we considered the fraction of hinge residues among drug-binding residues (*s/b*) and compared it to the fraction of hinge residues elsewhere in the protein, (*h-s)/(N-b*). The ratio of these two quantities, *e* = *s(N-b*)/*[b(h-s*)], provides a measure of the enrichment of hinge residues within drug-binding sites, as compared to other sites. As presented in Fig. 6*A* and *SI Appendix*, Table S2, an average enrichment of 4.156 is observed. The MAPK family is distinguished by an almost 20-fold enrichment. Yet, even after excluding MAPK, the enrichment averaged is 3.356 ± 0.875, i.e., the proportion of hinge residues is more than threefold enhanced among drug-binding sites compared to other regions.

From another perspective, if we examine the fraction of hinge residues among drug-binding residues, an average of 0.325 ± 0.104 is observed (Fig. 6*B*), i.e., about 1/3 of drug-binding residues act as hinges.

### Does the High Overlap Reflect the Evolution of Target Proteins to Optimize their Mechanochemical Behavior?

These results overall point to the fact that many FDA-approved drugs already bind hinge sites, even if they were not designed to interfere with the conformational mechanics of the target protein. This may be a consequence of the evolution of proteins to colocalize their chemically active and mechanically critical sites to enable the exchange of chemical and mechanical energies. This further begs questions about the role of the close neighborhood, or overlap, of hinge sites in the efficacy of these drugs, i.e., whether their ability to interfere with the protein dynamics adds to their efficacy.

The high propensity of hinge residues among drug-binding and/or functional sites invites attention to the significance of conformational mechanics as a determinant of function. It also calls attention to a strategy for designing inhibitors/activators of function: targeting mechanosensitive sites or hinges in the global modes, in addition to traditional targeting orthosteric sites or allosteric sites.

Global hinges mediate the soft modes. These modes are usually highly cooperative and robustly underlie the allosteric changes in conformations, or domain movements around an active site (49, 50), hence the effective targeting of global hinges by known drugs. Notably, high-frequency modes also involve residues that may transduce allosteric signals via tight interactions at centers of energy localization. Previous work showed that these residues tend to be evolutionarily conserved due to their role in stabilizing the fold (51, 52). However, they are often buried in the protein core and therefore not easily accessible for drug binding.

### A High Overlap was Attainable Even with a Single Mode.

**Fig. 7.**
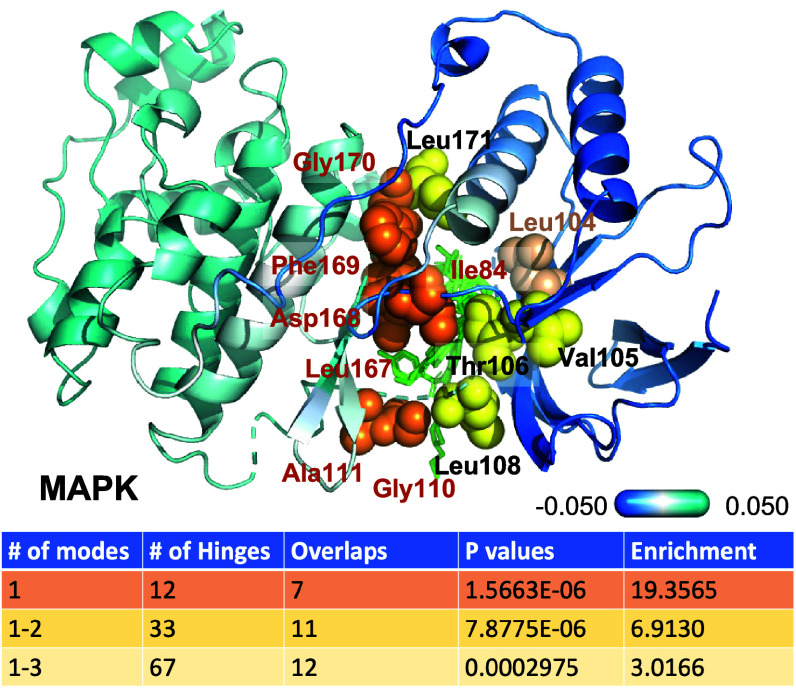
Results for MAPK14. The *diagram* shows the hinge residues from mode 1 (*orange*), 2 (*yellow*), and 3 (*wheat*) lining the drug-binding pocket. Their residue labels are *orange, black*, and *wheat*, respectively. A series of drugs (from multiple structures structurally aligned against the reference), are shown in *green sticks*. The ribbon diagram is colored by the normalized displacements along mode 1. The *Lower panel* lists the number of hinge residues in mode 1, 1-2, and 1-3, their overlap with drug-binding residues, corresponding hypergeometric scores, and enrichment at drug-binding sites.

Among the 20 protein families, six were distinguished by highly significant (< 10^−5^) *P* values: MAPK14 (p38a kinase), DNA polymerase (pol b), adenylate kinase (AK), peptidase S45 (PA), prostaglandin G/H synthase (COX2), and voltage-gated sodium channel (SCN5A). The high overlap observed in MAPK, pol β, and AK could be traced back to hinge sites associated with a single global mode intrinsically accessible to their structure, which overlapped with a significant number of drug-binding sites (*SI Appendix*, Table S2). As illustrated in Fig. 7, mode 1 of MAPK predicts 12 hinge residues, seven of which overlap with drug-binding residues (*orange spheres*). These include the DFG motif D168-G170 critically located between the ATP binding site and the kinase activation loop. Mode 2 adds four more overlaps, and mode 3 one more (*yellow* and *wheat spheres*, respectively). As shown in previous work, these modes enable the anticorrelated movements of the G loop and the substrate recognition site with respect to the activation loop and are therefore essential for allosteric communication between the ATP binding and substrate recognition sites (34). However, each mode also adds additional hinge residues that do not overlap with drug-binding residues (false positives) leading to higher *P* values or lower enrichment. A similar behavior is observed in AK: mode 1 yields 14 hits out of 20 predicted hinges. Mode 2 contributes another four while the total number of hinges is doubled. In either case, the highest enrichment is obtained with a single mode. Notably, mode 3 was the single most functional mode in pol β, as illustrated in *SI Appendix*, Fig. S4 (53).

### Not All Sites Constrained in Global Modes Serve as Hinges and not All Hinges Are Druggable.

Hinge sites are predicted by the GNM as structural elements significantly constrained in global modes. However, not all residues subject to minimal fluctuations in global mode shapes are hinges. Some may act as anchors/hinges between substructures that undergo opposite motions; others may be severely constrained because of their localization in a densely packed environment rather than bridging between oppositely moving elements. Such regions are often manifested by long stretches of sequentially contiguous residues forming rigid-like clusters in the 3D structure. They do not serve as hinges, except for the residues at the boundaries of these blocks, which may allow for flexing of the adjoining structural elements. Further, GNM-predicted hinge sites close to chain termini may not be good target sites, as the effect of the drug would be dissipated locally without being transmitted to the bulk of the protein. Therefore, we adopted a number of filters in our algorithm to automatically select from among minima in the global modes, those sites that are likely to serve as druggable hinges (presented in, *SI Appendix, Supporting Methods*). The method is extremely efficient: Hinge sites for a protein of ~ 1,000 residues are identified within seconds. Yet, inspection of predictions, especially residue specificities at hinge sites, which the GNM does not consider, would be recommended for accurate predictions or interpretations of results in case studies.

### A New Strategy for Modulating Function: Targeting Mechanosensitive Sites.

Drugs are usually known to target chemical (e.g., catalytic) or physical (e.g., substrate-binding) sites. This is how orthosteric and allosteric sites interfere with the target protein’s function, respectively. On the other hand, many proteins operate as mechanochemical entities, and numerous studies highlight the role of conformational mechanics in enabling function. The current study suggests that mechanosensitive sites, such as hinge sites, could also serve as target sites. Such considerations may also help accelerate drug discovery efforts. For example, druggability simulations or docking campaigns often suggest multiple sites for drug binding. Hinge sites that may be efficiently identified using our tool could be prioritized among those druggable sites.

Notably, our analysis shows that many hinge sites are already targeted by drugs. Yet, the study reveals many other hinge sites, not targeted by drugs. While these were assumed here to be false positives in our statistical evaluations (of *P-*values and enrichments), we noted that some of them coincided with functional sites (*SI Appendix*, Table S3) and could potentially serve as target sites. A systematic study of druggable sites in kinases, for example, pointed to many regulatory hot spots that could be considered for allo-targeting (53). An advantage of targeting a hinge site might be the ability to interfere with a specific movement relevant to a particular function, rather than interfering indiscriminately with the protein overall activity. In this respect, hinge sites could be comparable to allosteric sites as they can modulate selective conformational changes. In particular, the evolutionarily conserved, or coevolving, residues at hinge sites may be critically important to function. These could be explored as alternative drug-binding sites, possibly in combination with orthosteric or allosteric sites. In view of the proven utility of combination therapies in complex diseases (54), such polypharmacological interventions may prove useful for alleviating drug resistance.

## Constructing Ensembles From Reference Structures Based on Homology and Structural Criteria

The ensembles of structural homologs were identified using *SignDy* (39) and the Dali server, based on following criteria: For relatively small proteins (*N* ≤ 550), sequence identity threshold was set to > 85%, together with z-scores > 10, and RMSD with respect to reference was restricted to < 2 Å; for larger proteins, the criteria were sequence identity > 95%, z-scores > 10, and RMSD > 1 Å.

## Identification of Hinge Residues and their Overlap with Drug-Binding Residues

We used the procedure described in the Approach and *SI Appendix* for identifying hinge sites and drug-binding sites. The code for predicting and evaluating hinges sites is accessible online (https://github.com/HaotianFrankZhang/Allocate-Protein-Hinges.git). All drugs included here were approved drugs, except for the molecules in *SI Appendix*, Table S4 included for AK and pol β to increase their sample size.

## Supplementary Material

Appendix 01 (PDF)

## Data Availability

All study data are included in the article and/or *SI Appendix*. The codes developed for this study and the generated data are deposited in GitHub (55).
