# Supplementary material for: Global hinge sites of proteins as target sites for drug binding
Source: Proc Natl Acad Sci U S A. 2024 Nov 25;121(49):e2414333121. doi: 10.1073/pnas.2414333121 (PMC11626116; doi:10.1073/pnas.2414333121)
Supplement: Supplementary file 1 — Appendix 01 (PDF) [file pnas.2414333121.sapp.pdf]

PNAS

Supporting Information for
Global hinge sites of proteins as target sites for drug binding

Haotian Zhang,[1] Mert Gur,[1] and Ivet Bahar[1,2]

[1]*Department of Computational and Systems Biology, School of Medicine, University of Pittsburgh, Pittsburgh, PA 15261, USA*
[2]*Laufer Center for Physical and Quantitative Biology and Department of Biochemistry and Cell Biology, School of Medicine, Stony Brook University, New York 11794, USA*

Corresponding author: Ivet Bahar
Email: bahar@laufercenter.org

This PDF file includes:

- Supplementary methods
- Figures S1 to S4
- Tables S1 to S4
- SI References

Supporting methods

Selection of dataset proteins. The selection of the dataset of proteins analyzed in the present study consisted of two steps: First we selected the 'major' families of proteins, which are known to be the most common drug targets. These were based on an extensive mapping of molecular drug targets (1), which pointed to 'major' families of proteins targeted by drug molecules. Notably, GPCRs, ion channels, nuclear receptors and kinases were reported to account for 44% of all human protein targets, and 70% of ChEMBL-listed drugs targeted these four 'major families'. These were considered, together with eight other major families **(Table S1**) to construct our dataset of 20 protein families (**Table 1**), which included 1-4 members (protein families) from each 'major family' (**Table S1**). A few criteria distinguishing multiple families within the same major family, in addition to diversity of functions and folds, are written in **Table S1** footnote. As a second step, we generated an ensemble of proteins for each of the 20 protein families using our *SignDy* module (2) of the interface *ProDy* (3), in conjunction with the Dali server (4). SignDy has been developed for systematic retrieval and analyses of ensembles of structural homologs from the PDB to enable efficient statistical analysis of sequence, structure and dynamics properties. The method for selecting these ensembles, which uses the PDB and analyzing ensembles of structural homologs) is presented in the Main text, under **DATA, Materials, Methods and Software Availability.**

Selection of modes. As explained in the text, hinge sites were determined based on the global modes of motion predicted by the GNM. Up to three slowest modes, $1 \le k \le 3$, were included to ensure a cumulative fractional variance s of ≥ 1/3. The fractional variance contributed by *n* modes is calculated as

$$s(n) = \sum_{k=1}^{n} 1/\lambda_k \Big/ \sum_{k=1}^{N-1} 1/\lambda_k \qquad (1)$$

where λ_k designates the kth eigenvalue of the Kirchhoff matrix Γ of the GNM. The Kirchhoff matrix is a symmetric *N x N* matrix for a protein of *N* residues, composed of the following elements

$$\Gamma_{ii} = \{z_i \quad if\ 2 \le i \le N-1 \quad \text{and} \quad \Gamma_{ij} = \begin{cases} -1 & if\ r_{ij} < r_{cut} \\ 0 & otherwise \end{cases} \qquad (2)$$

The eigenvalue decomposition of Γ yields *N-1* non-zero eigenvalues ($\lambda_1 < \lambda_2 < \ldots. < \lambda_{N-1}$) scaling with the frequency of individual modes, λ_1 corresponding to the slowest (or softest or lowest frequency) mode of motion. Soft modes usually encompass the entire structure, hence their qualification as global modes. Each mode refers to a collective direction of motion, represented by the kth eigenvector $\boldsymbol{u}_k$ of Γ. The selected modes, which account for 1/3 of the complete spectrum of motions accessible under equilibrium conditions, also represent the most cooperative motions which are uniquely encoded by the protein family architecture. While this is a small fraction of the total number (*N*-1) of GNM modes, it is sufficient to identify approximately *N*/10 hinge residues, whose possible overlaps with drug-binding residues were subsequently examined.

Identification of hinge sites. The hinge residues in mode k are deduced from the normalized displacement of residues along the kth mode axis, also called the kth *mode profile*. The kth mode profile is obtained by plotting the $1 \leq i \leq N$ elements $\boldsymbol{u}_k^{(i)}$ of $\boldsymbol{u}_k$ each element as a function of residue index i. Those elements lying at the crossover between negative and positive movements are the hinge residues in mode k. The residues at the crossover region of *mode k* are selected using the following protocol:

(a) The hinge residues were required to cross a narrow band near the zero line ($y = 0$) of the mode shape (elements $u_k^{(i)}$ of $\boldsymbol{u}_k$ plotted as a function of residue index i). If one or more residues entered the band from one side, and left from the *same* side without crossing the band, these were not considered as hinge residues.

(b) The band width was equal to $\pm cN^{-1/2}$. For a given protein of N residues, the band width was selected to be proportional to the average size $\langle u_k^{(i)} \rangle = N^{-1/2}$ of the elements of (normalized) eigenvectors; and the proportionality constant c was sufficiently small to restrict hinge residues to those undergoing minimal translational movements, if any. The value of $c = 1/15$ was adopted, which confined the hinge sites to a narrow band and yielded approximately $N/10$ hinge residues for a protein of N residues.

(c) If three or four residues were observed to fall within the band and exhibited opposite signs at the two terminal residues (i.e. cross the $y = 0$ line), they were all classified as hinge residues.

(d) If the band region contained $x = 5$ or more residues, we selected as hinge residues the consecutive pair closest to the zero line, plus $(x-2)/4$ additional residues sorted from smallest to largest absolute value.

(e) We excluded the N- and C-terminal N/20 residues unless these segments harbored known active sites (as in DHFR, AKR1C3, PA, and SCN5A).

Mapping Drug-Binding Sites Through Ensemble Analysis. We identified drug binding residues as those making atom-atom contacts within 5Å with the drugs. To this aim, for each of the 20 families listed in **Table 1**, we considered all the *m* proteins and checked whether they contained FDA-approved drugs, and if so, we identified and compiled the corresponding drug-binding residues, which resulted in *b* drug-binding residues. The selected set of drugs and drug-binding sites were subjected to multiple filters: First, to check whether a given small molecule was an approved drug or not, we screened the identified small molecules against the approved drugs in DrugBank (7). Second, we considered the 281,777 biological assemblies resolved in the presence of one or more small molecules, available in the PDB as of 4/2022, and those small molecules found over 500 times in different assemblies were excluded as common solvents or lipids (and not drugs), according to the IUPAC nomenclature. Third, we selected compounds with molecular weights varying in the range 100 to 1000 g/mol. This process resulted in 1,018 drugs for our 20 protein families.

The PDB biological assembly database (8) was used to verify that the structures used represented the biologically functional state of the protein, not substructures, building blocks or asymmetric units. Drug-binding residues were determined by multi-sequence alignments of the m proteins in each family and validated against superimposed crystal structures. For AK, we included six additional ligands, and for pol β, the nucleotide analogs listed in **Table S4**, due to the lack of a sufficient number of crystal structures complexed with drugs.

Supporting figures

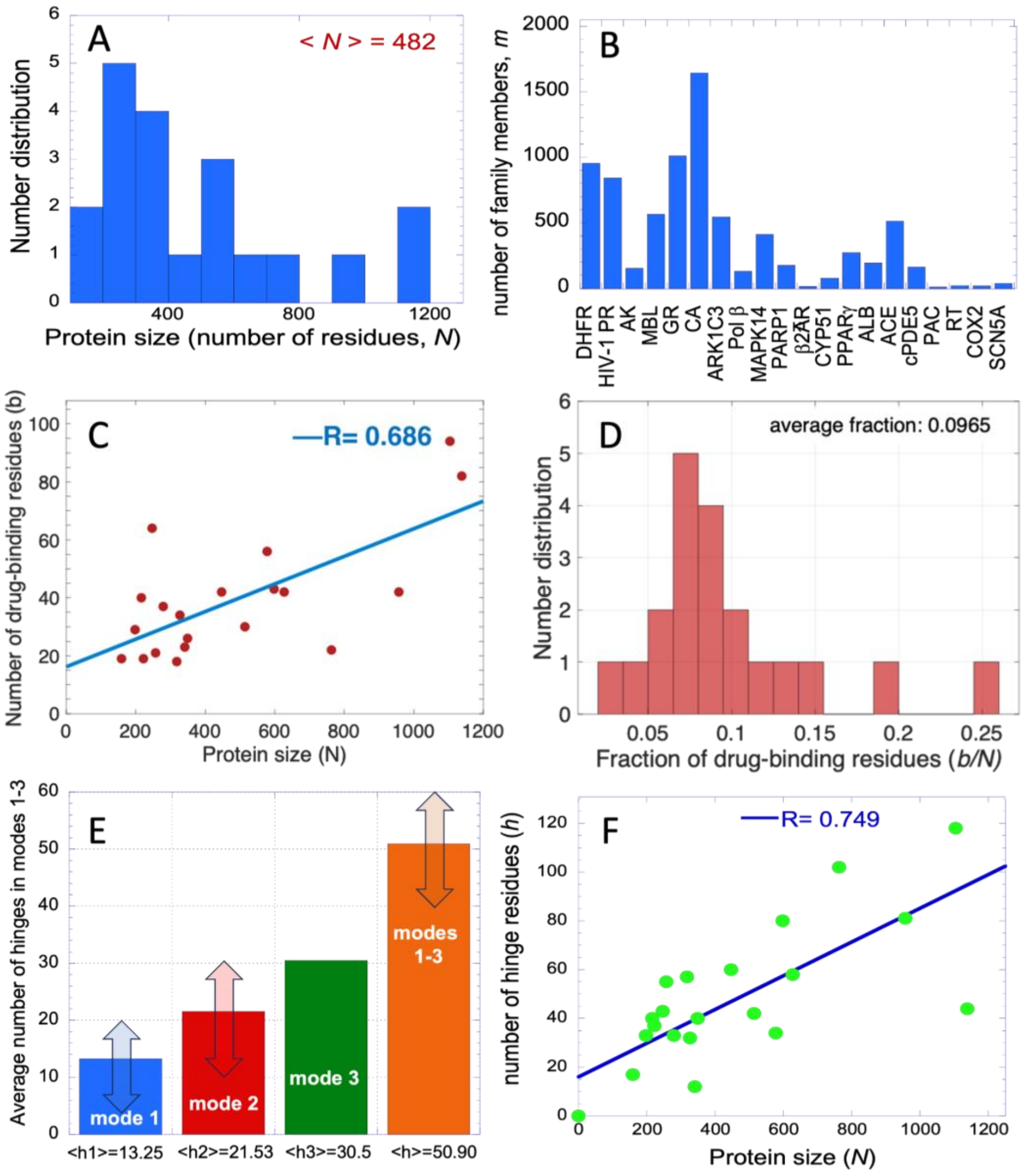

Fig. S1. Characteristics of the sets of drug-binding proteins examined in the present study. (A) Distribution of sizes (represented by the number of residues of the representative member in each ensemble); (B) Membership of each family, i.e. the number of structural homologs (and PDB structures) that were included in analyzing each family. The abscissa lists the name of the representative member, ordered by N, from small to large. See Table 1 for the full names and corresponding UNIPROT families. (C) Number of drug-binding residues (b) corresponding to each family, plotted as a function of the protein size. (D) Histogram of the fraction of drug-binding residues in each of the 20 examined cases. (E) Average number of hinge residues detected in mode 1, mode 2, mode 3, and modes 1-3. The averages are written along the abscissa, and the arrows indicate the standard deviations. Note that some of the hinge residues in different modes overlap, hence the smaller value of the total (of modes 1-3) compared to the sum of the hinges in individual modes. (F) Increase in the number of hinge residues in the global modes with increasing size of the protein.

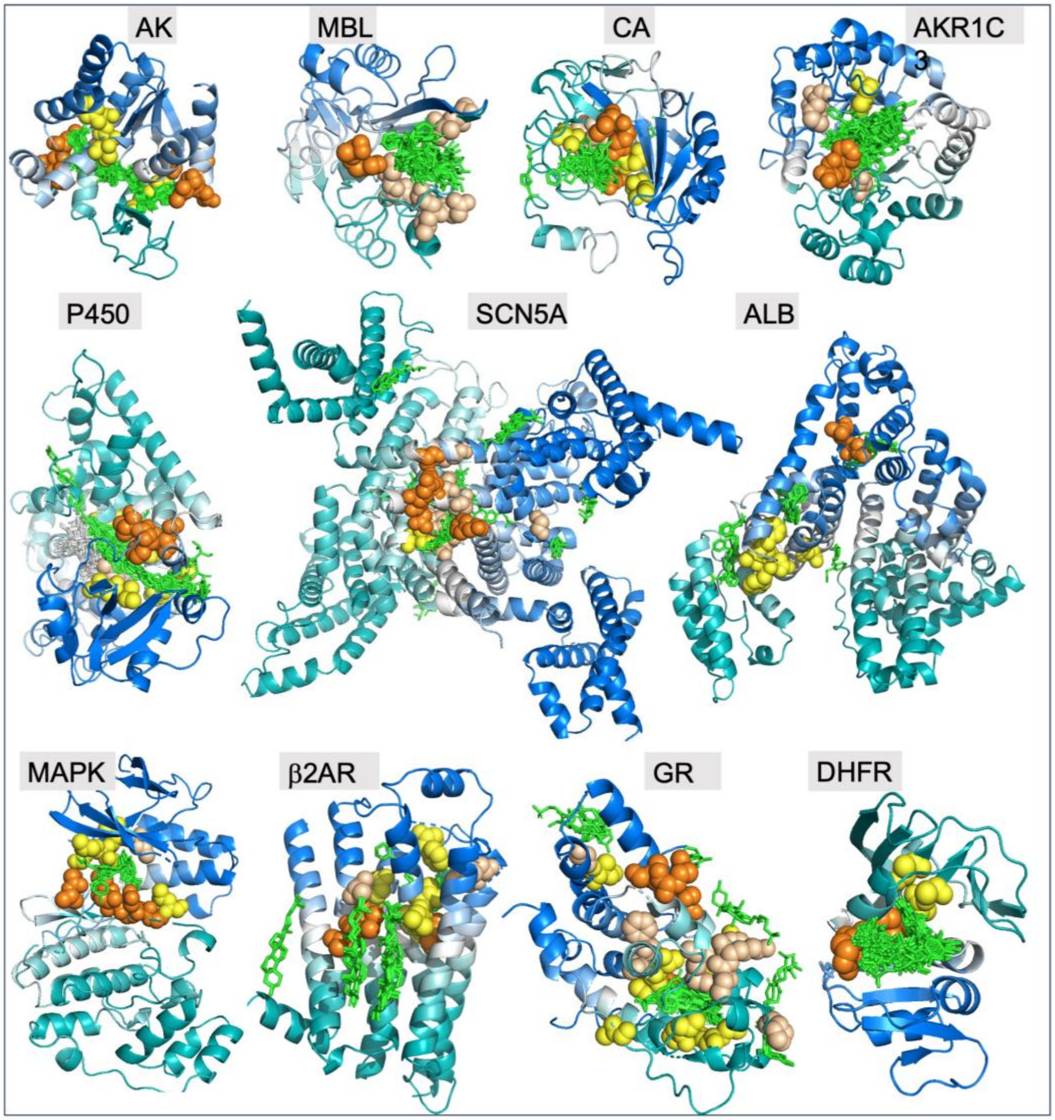

Fig. S2. Position of hinge sites and drug binding pockets shown for AK, MBL, CA, AKR1C, P450, SCN5A, ALB, MAPK, β2AR, GR, and DHFR. Position of hinge sites from mode 1 (*orange spheres*), mode 2 (*yellow spheres*), and mode 3 (*wheat spheres*) lining the drug-binding pocket. A series of drugs (from multiple structures) are displayed in *green sticks*, superposed after structural alignment of the corresponding PDB structures. The ribbon diagrams are color-coded based on the direction of the normalized displacements of residues along mode 1 axis, *blue* and *cyan* regions referring to movements in opposite directions, and hinge regions are in *white/gray*.

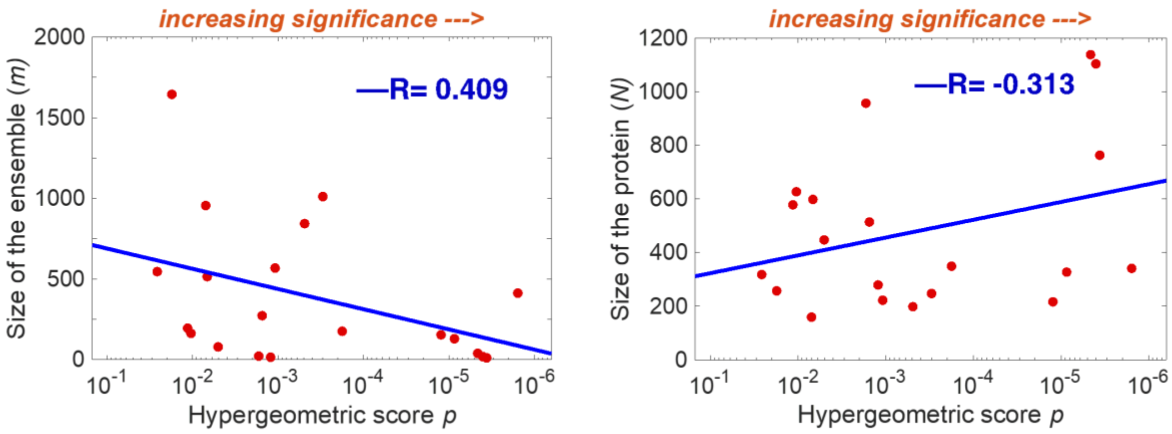

Fig. S3. Weak dependency of hypergeometric scores on the ensemble sizes (*m*) and on the protein sizes (*N*). Correlation between the hypergeometric score (*p*) and the size of the ensemble (*m*) (*left panel*) and the size of the protein (*N*) (*right panel*) are displayed for all 20 families of target proteins. The *left panel* shows a negative correlation (linear regression, R = -0.409) between the hypergeometric score and the ensemble size, while the *right panel* shows a small positive correlation (R = 0.313) between the hypergeometric score and protein size.

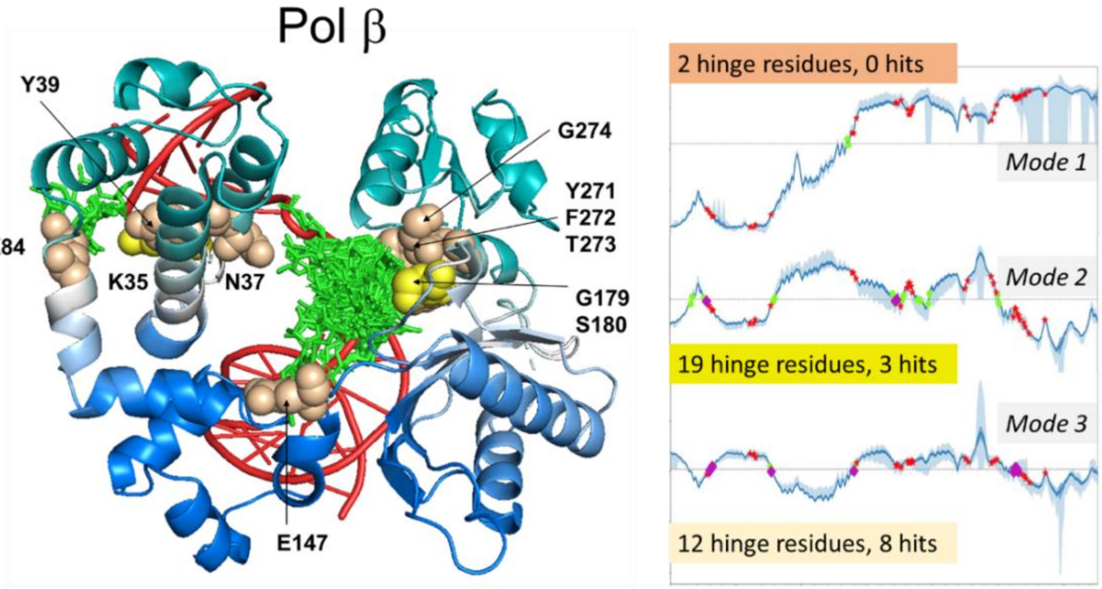

# of modes	# of Hinges	Overlaps	P values	Enrichment
1	2	0	1	0
1-2	19	3	0.3149	1.6158
1-3	32	11	8.6409e-5	4.5140

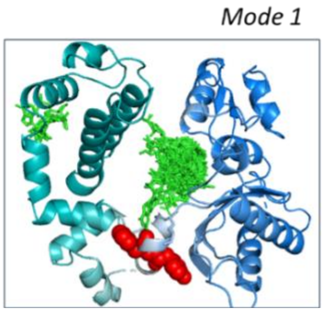

Fig. S4. Results for Pol β. The *top left panel* shows the position of hinge sites from *mode 2* (*yellow spheres*) and *mode 3* (*wheat spheres*) lining the drug-binding pocket. Reside labels are in black. A series of drugs (from multiple structures) are displayed in *green sticks*, superposed after structural alignment of the corresponding PDB structures. The ribbon diagram of Pol β is color-coded based on the displacements of residues along *mode 2.* The DNA is colored red. The *top right panel* shows the normalized distributions of residue displacements along the axes of global modes 1 (*top*), 2 (*middle*), and 3 (*bottom*) predicted by *SignDy*. The solid curve in each panel represents the average mode shape, and the *light blue shade* displays the standard deviation from the mean profile based on the examined 129 structural homologs. The position of the hinge sites (*green dots*), drug-binding sites (*red stars*) are shown along the curves, and the overlapping residues between the two subsets (*magenta diamonds*) are labeled. The *lower left panel* shows the number of hinge residues in *mode 1, modes 1-2* and *modes 1-3,* and corresponding overlaps with drug-binding sites, hypergeometric scores, and enrichment of hinge residues at drug-binding sites. The *lower right panel* shows the position of hinge sites from *mode 1* (*red spheres*) near the drug-binding pocket (drugs in *green sti*cks), but not overlapping with drug-coordinating residues.

Supporting tables

Table S1. Major families of drug targets [*], and corresponding members (protein families) included in our dataset (Table 1)

Major families of drug targets	Selected family in **Table 1**	
	Index	Acronym
1. GPCR	11[a]	β2AR
2. Nuclear receptor	5, 13[b]	GR, PPARγ
3. Ion channel	20[c]	SCN5A
4. Kinase	9, 3[d]	MAPK14, AK
5. Reductase	1, 7, 19[e]	DHFR, AKR1C3, COX2
6. Protease	2, 15 [f]	HIV-1 PR
7. Hydrolase	4, 15, 17, 18[f]	MBL, GR, PA, RT
8. Transferase	3, 8, 10, 18[f]	AK, Polβ, PARP1, RT
9. Phosphodiesterase	16	cPDE5
10. Lyase	6,8[g]	CA, Polβ
11. Cytochrome P450	12 [h]	P450
12. Transporter	14[i]	ALB

[*] *from Figure 1 of* Santos *et al.*, A comprehensive map of molecular drug targets. *Nat Rev Drug Discov* **16**, 19-34 (2017).

[a] *Rhodopsin-like;* [b] *different oligomerization states;* [c] *voltage-gated ion channel with high molecular weight (N = 1138); high structural flexibility;* [d] *AK is also classified as a transferase;* [e] *diversity of functions among reductases;* [f] *different (extreme) ensemble sizes and structural variations within the ensembles,* [g] *DNA-bound structure;* [h] *structurally diverse (relatively high RMSD), despite small size;* [i] *structurally diverse ensemble*

Table S2. Number of drug-binding and hinge residues (*b* and *h*), their overlap (*s*), and enrichment of hinges ay drug-binding sites compared to other sites

Protein [(1)]	*N*	*b*	*h*[(2)]	*s*	Enrichment [(4)]	s/b x 100 [(5)]
DHFR	159	19	17	6	4.0191	31.58%
HIV-1 PR	198	29	33	12	3.3300	41.38%
AK	216	40	40	18	3.6000	45.00%
MBL2	222	19	37	9	3.4342	47.37%
GR	247	36	40	12	3.1604	33.33%
CA	257	21	55	9	2.1988	42.86%
AKR1C3	318	18	57	7	2.3333	38.89%
Polβ	327	34	32	11	4.5140	32.35%
MAPK14	341	23	12	7	19.357	30.43%
PARP1	349	26	40	10	4.1410	38.46%
b2AR	279	37	33	11	3.2703	29.73%
P450	447	42	60	12	2.4107	28.57%
PPARg	514	30	42	8	3.7961	26.67%
ALB	578	56	34	8	2.8681	14.29%
ACE	598	43	80	12	2.2777	27.91%
cPDE5	627	42	58	9	2.5583	21.43%
PA	763	22	102	12	4.4909	54.55%
HIV-1 RT	957	42	81	10	3.0684	23.81%
COX2	1104	94	118	25	2.8884	26.60%
SCN5A	1138	82	44	13	5.4005	15.85%
**Average**					**4.156**	**32.25%**

[1]*See full names in* ***Table 1****; N is the number of residues, b is the number of drug-binding residues, h is the number of hinge residues, sis the overlap between b and h subsets*

[2]*Hinges based on 3 slowest modes, except for 5 cases that met the criteria of 33% variance with 1 mode (MAPK14) or 2 modes (DHFR, AK, ALB, PARP1)*

[3]*Sequence coverage threshold is decreased for PA from 95% to 90% to increase sample size.*

[4]*enrichment is calculated by the ratio of (i) the fraction of hinges within binding sites (s/b) to (ii) the fraction of hinges at other sites, (h-s)/(N-b), i.e., the ratio (s/b)/[(h-s)/(N-b)] = s(N-b)/[b(h-s)]*

[5]*percentage of hinge sites among drug-binding sites*

Table S3. Overlaps between hinges and drug-binding sites, and comparison with active sites*(1)*

Protein acronym	Overlaps (hinge residues co-localized with drug-binding sites) (1)			Active / functional sites (2) including additional hinge sites (3)	Total # of overlaps
	mode 1	*mode 2*	*mode 3*		
DHFR	**I5**, K32, T35	**A6**, **A7**, **T113**	N/A	Chemical site: D27, W30, Y111, **T113**<br>Substrate binding: **I5**, **A6**, **A7**, D27, G43, R44, H45, T46, R57, **T113**<br>NADP+ binding: **A6**, **A7**, I14, G15, M16, E17, N18, A19, L62, S63, S64, Q65, V78, I94, G95, G96, G97, R98, V99	6
HIV-1 PR	A: G49, I50, G51; B: G51, G52	A: T31, I94; B: T31, V32	A: D30, B: D30, F53 *(A: G49, I50, G51, B: G52; A: T31, I84, B: T31)*	Chemical site: D25, T26, and <u>*G27*</u> *(mode 3)*	12 + 1
AK	L8, P9, **G12, K13, G14**, Q16, I53, **E57**, **L58**, N175, G197, **Q199**, D200, I201	S30, **G85**, **F86**, R123	N/A	ATP binding: G10, A11, **G12**, **K13**, **G14**, T15, R127, **Q199**<br>AMP binding: T31, R36, **E57**, **L58**, V59, **G85**, **F86**, P87 R88, Q92, R160, R171	18
MBL2	**H116**, A117	-	A68, V69, **C198**, V200, D207, L208	Chemical site: **H116**, H118, D120, H179, **C198**, H240; Substrate binding: <u>*K201*</u> *(mode 3),* A210	9 +1
GR	W600, R655, L656, S724	L544, M560, L563, N564, M604, A605, I694, N731	F606, A607, P637, C643, K644, H645, M646, G698	(AP2, p166 interaction sites): K579, R585, D590, L745, R746, L748, L749, D750, D752	21
CA	L91, **Q92**, E106	S65, **H94**, V207	**H119**, V143, W209 *(0; H94)*	Chemical site: H64, **H94**, H96, H119<br>Activator binding of: **Q92**<br>Zn binding: **H94**, H96, **H119**<br>Substrate binding: L198, T199	9
AKR1C3	**F306, N307**	W86	P119, M120, **Y216**, **S217** *(0; W86)*	Chemical site: Y55, **C-term;** NADP+ binding: T23, Y24, <u>*D50*</u> *(mode1),* S166, N167, <u>*Q190*</u> *(mode1),* **Y216**, **S217**, <u>*A218*</u> *(mode3), <u>L219</u> (mode3),* G220, S221, Q222, K270, S271, Y272, R276, I277, R278, Q279, N280; substrate-binding; H117; ligand recognition/ product release: W227, F306	7 + 4
Polβ	-	**K35**, G179, **S180**	N37, Y39, K84, E147, Y271, F272, T273, G274	Chemical site: Lyases K72, **K35**, K68<br>dNTP binding: R149, **S180**, R183, G189, D190, D192; ion binding: K60, L62, V65, T101, V103, I106, D190, D192, D256	11
MAPK14	I84, G110, A111, L167, **D168**, F169, G170	N/A	N/A	Chemical site: **D168**<br>ATP binding site: V30, G31, S32, G33, A34, Y35, G36, S37, V38, K53	7

PARP1	I879, A880, G894	E763, D766, **H862**, **G863**, L877, **R878**, **S904**	N/A	Chemical site: E988<br>NAD+ binding site: **H862**, **G863**, S864, G871, **R878**, **S904**	10
b2AR	L80, V81, **T118**	V114, L115, V117, F193, **Y316**	A85, Y199, A200	Carazolol binding (S): D113, S203, N312; Timolol binding: D113, **T118**, N293, N312, **Y316**	11
P450	**F78**, **M79**, G175, T176	G257, H258, **I323**, L324, H430, S431, **V434**	**L321**	Chemical site: Y76, **F78**, **M79**, F83, F89, K97, M99, H101, S252, F255, H259, T260, **L321**, **I323**, M433, **V434**<br>Heme b Binding: *Q72 (mode 3*), Y76, K97, R326, H392; Fe Binding: C394	12 +1
PPARg	-	A: A292, I326, B: A292	A: L330, B: C285, R288, I326, Y327	S245 (phosphorylation), *K367 (mode 2, chain B)* (SUMOylation)	8 +1
ALB	L346, L349	Y401, K402, F403, Q404, L407, N429	N/A	Asprin-acetylated lysine: K199<br>Binding site bilirubin IXalpha: K240<br>Metal binding: E6, D13, H67, *E244 (mode 2)*, H247, D249, E252, *D255 (mode 2),* D259	8 +2
ACE	S339, A340, F375, E503, Y504	K102, L103, Y507	W204, D206, H394, **E395**	E368, H497<br>Binding site Zn: H367, H371, **E395**	12
cPDE5	-	A: **D764**, L765, A767, G819, F820	B: Y612, **H613**, L725, V782	**H613 (A, B)**<br>Binding site AMP: H613, N614, W615, R616, H617, D654, **D764**, Q817<br>Binding site ZN: H617, H653, D654, **D764**	9
PA	A: S149, **B: S1**, F71	A: M142, B: T68, A69, A241, N388(B: S1)	A: R145, F146, B: F24, S386	**S1(B)** Ca Binding: E152(A), *D73(B) (mode 1)*, V75(B), D76(B), P205(B), D252 (B)	12 +1
HIV-1 RT	-	-	A: T107, V108, V179, I180, Y181, Q182, Y183, L187, 188, V189	A: D110, D185, D186, D443, E478, D498, D549 Binding site, Mg+; catalytic; for reverse transcriptase activity: D110, D185, D186; Mg++ binding site; catalytic; for reverse transcriptase activity: D443, E478, D498, D549	10
COX2	-	A: L117, Q192, F209, L352, S353, N375, K532, G533, B: M113, Q192, F209, H351, ;352, N375, G533	A: I345, Y348, V349, H351, L534, G536, B: I345, Y348, V349, L534 (0; A: Q192, L352, G533, B: Q192, G533)	Chemical sites: H207, Y385 (A, B)<br>Binding site for substrate: R120(A, B), I335(A, B); Binding site for Fe of heme: H388 (A, B); site aspirin-acetylated serine: S530(A, B)	25
SCN5A	F945, A1332, **N1474**, N1476, Q1477	I1773	L410, G834, I1336, I1468, G1469, I1471, Y1769	(IFM motif): **N1474**, F1475, G1478, I1487, F1488, M1489, T1490, E1491, Q1493, K1495, L1503, K1507, P1508, Q1509, N1661*, E1775 (mode 2)*<br>(DEKA motif): D373, E901, K1421, A1713; (S4 segments): K1643, R1646	13 +1

*[(1)] Overlaps, also termed hits, refer to the s residues overlapping between computationally predicted hinge residues and experimentally observed drug-binding residues. Hinge residues are based on mode 1, 2, or 3, except for the cases annotated as N/A. N/A refers to modes that were not included because the contribution of first 1 or 2 modes satisfied the required 1/3 cumulative variance. The symbol '-' indicates no overlaps. Residues in bold face also overlap with active site (or functional) residues reported in the column 5. Residue numbers refer to the reference proteins (PDB files) written in the first column (and in **Table 1**).*
[(2)] Functional sites reported in UniProt or in the literature (e.g., catalytic, ligand-binding, ion-binding). The entries in boldface are identified as hinges and drug-binding residues. The same residues are written in bold face in columns 2-4. [(3)] Residues underlined (and in blue) are other hinges identified in modes 1-3 (as noted in parentheses), which do not overlap with drug-binding sites.

Table S4. Additional ligands included in the analysis for AK and Pol β

Protein	Additional ligands' acronyms
AK	AP5, ADP, AMP, ANP, C5P, UDP
Pol β	VT7, B7J, GFF, VC8, BPI, 8PI, FHG, 2RW, GOA, ACT, FDV, DDG, GTP, FHA, YQS, 2DA, QPJ, 2DT, FDY, 2TM, XC5, FCJ, 8DG, H84, VT8, FF4, 1GC, CTP, TTE, 8CP, MDN, F3C, TTP, ZAN, 1FO, DZ4, 61C, UTP, EDO, PEG, FFJ, APC, VA6, SFV, D3T, DCT, 1RZ, VA4, F2A, V3A, AZT, O2C, VT6, DAD, B7A, GRC, IMD, HGV, DTP, TFF, G1M, ATP, EJH, CAC, VA7, 1S0, PG4, 1RY, FF7, 42E, DGI, N6T, DCP, F3A, 6CF, DGT, G2C, DUP, G1C, GKS, 1FZ, DOC, OXL, XG4, 1FQ, G2M, GFH, DJJ, VA5, GGH, GBR, FDJ, SGT, 8GT, VC9, GFM, 4BD, B7P, 2KH, 0KX, FMT, C6T, VC6, GFC

SI References

1. R. Santos *et al.*, A comprehensive map of molecular drug targets. *Nat Rev Drug Discov* **16**, 19-34 (2017).
2. S. Zhang, H. C. Li, J. Krieger, I. Bahar, Shared Signature Dynamics Tempered by Local Fluctuations Enables Fold Adaptability and Specificity. *Molecular Biology and Evolution* **36**, 2053-2068 (2019).
3. S. Zhang *et al.*, 2.0: increased scale and scope after 10 years of protein dynamics modelling with Python. *Bioinformatics* **37**, 3657-3659 (2021).
4. L. Holm, Dali server: structural unification of protein families. *Nucleic Acids Research* **50**, W210-W215 (2022).
5. T. Haliloglu, I. Bahar, B. Erman, Gaussian dynamics of folded proteins. *Phys Rev Lett* **79**, 3090-3093 (1997).
6. A. Rader, C. Chennubhotla, L.-W. Yang, I. Bahar, The Gaussian Network Model: Theory and Applications. *Normal Mode Analysis. Theory and Applications to Biological and Chemical Systems* (2006).
7. D. S. Wishart *et al.*, DrugBank: a knowledgebase for drugs, drug actions and drug targets. *Nucleic Acids Res* **36**, D901-D906 (2008).
8. H. M. Berman *et al.*, The Protein Data Bank. *Nucleic Acids Res* **28**, 235-242 (2000).
